# Experimental Investigation on the Residual Stresses in a Thick Joint with a Partial Repair Weld Using Multiple-Cut Contour Method

**DOI:** 10.3390/ma11040633

**Published:** 2018-04-20

**Authors:** Chuan Liu, Chunjing Wang, Xiaohua Cheng, Yi Yan, Jiawei Yang, Yuhang Guo

**Affiliations:** 1 Provincial Key Lab of Advanced Welding Technology, Jiangsu University of Science and Technology, Zhenjiang 212003, China; chunjing1994@126.com (C.W.); yiyan_lengduo@126.com (Y.Y.); young10402@163.com (J.Y.); guoyuhang@just.edu.cn (Y.G.); 2 China Nuclear Industry Huaxing Construction Company Limited, Nanjing 21000, China; chxh9361@aliyun.com

**Keywords:** repair weld, residual stress, multiple-cut contour method, thick welded specimen, superposition

## Abstract

The stress distributions in a thick welded specimen with a partial repair weld were measured with the three-cut contour method. The longitudinal stress maps in the original weld and the repair weld were obtained and the transverse stress map at the weld centerline in the original weld was acquired. The difference between the longitudinal stress in the partial repair weld and that in the original weld was investigated. Results show that the longitudinal stress increases significantly within the entire repair region with a peak tensile longitudinal stress close to the yield strength of weld material; and the longitudinal stress in the region above the repair weld decreases distinctly after repair; the introduction of the partial repair weld does not affect the stress distribution trend in the original weld (whether it is beyond or above the repair weld), and it has a slight effect on the tensile stress distribution width in the repair region.

## 1. Introduction

Welding residual stresses, developed due to localized heating and non-uniform cooling accompanied with steep thermal gradients that arise in the weld zone, have been known as one of the most critical factors in weldments. The detrimental effects of welding residual stresses on the structural integrity, fatigue performance, service security, and remaining life of the welded structures are significant [1]. Welded components are usually repaired during manufacture or in-service after removal of defects or degraded material. Repair welds can influence the magnitude and distribution of the as-welded residual stresses in the weldments. The stress distribution and variation in repair welds have attracted much attention during recent decades. For example, Dong et al. [2,3] found that the weld repairs typically increase the magnitude of transverse residual stresses along the repair compared with the original weld and that the shorter the repair length the greater the increase in the transverse stress. Recently, Song and Dong [4] suggested that a weld repair should be designed as long as possible, as narrow as possible, and as shallow as possible.

Experimental evaluation is the essential method to investigate the welding residual stress and also provide sufficient information to verify the numerical simulation model. The methods to experimentally determine the surface stress, such as the hole-drilling method and X-ray diffraction (XRD) method, are available to measure the welding stress before and after repair. For example, Zeinoddini et al. [5] measured the residual stresses in single/double and partial/full repair welds in offshore pipelines with the hole-drilling method. It was found that the repetition of repair welding in same area influenced the stress magnitude and distribution especially in areas close to the weld centerline. Veiga et al. [6] investigated the influence of the repair procedure on the evolution of residual stress distribution in 11 mm-thick butt welds with the XRD method. They found that the repair welds caused a decrease in magnitude of the initial longitudinal residual welding stresses, and an increase of the transverse residual stress magnitude in tension at points within the repair length, and in compression at points outside the repair length.

The repair weld needs to fully or partially remove the initial weld material, the internal stress distribution after repair could be much different from the original condition and the measurement of surface stress is not enough to investigate internal stress variation. Therefore, the methods with the ability of measuring the through-thickness stress, such as the deep-hole method and neutron diffraction (ND) method, are adopted to evaluate the through-thickness stress variation before and after repair. For example, George and Smith [7] measured the through-thickness residual stress profiles before and after introducing deep and shallow part-circumferential weld repairs into a 37 mm thick stainless steel cylinder using the deep-hole drilling method. They found that the membrane and bending components of the in-plane residual stresses were generally increased when weld repairs were introduced. Bouchard et al. [8] investigated the residual stresses in a stainless steel pipe girth weld containing long and short repairs with the deep-hole method, ND method, and surface hole-drill technique. It was indicated that the residual stresses at mid-length of the heat affected zone of the short repair were found to be higher than those in the long repair.

The research results obtained by Dong et al. [2], Song and Dong [4], and Bouchard et al. [8] demonstrated that the residual stresses in repair welds typically exhibit strong three-dimensional (3D) features, depending on repair geometries. Therefore, a 2D or 3D residual stress map is more useful to understand the stress distribution and variation in repair welds. The deep-hole method can only get the through-thickness stress at limited locations. Diffraction techniques might seem preferable to get the 2D or 3D stress distributions within the sample without affecting the integrity of the specimen under investigation. However, a number of limitations inherent to both neutron and synchrotron diffraction techniques exist, including the limited specimen thickness and grain size, high cost, requirement of stress-free reference specimens, sensitivity to microstructure, and equipment not being readily available [9].

The contour method (CM) is a promising relaxation method to get the full 2D stress map on a plane of interest [10]. In addition, it is insensitive to microstructure variation (weld/parent metal), the sample thickness, and the required equipment is readily available [9]. The CM has been used to achieve full 2D stress maps in specimens with different welding methods including arc welding [11], variable-polarity plasma-arc welding [12], friction welding [13,14,15,16], and electron beam welding [17]. Comparisons with the ND measurements indicate that the contour method can accurately measure through thickness residual stresses [18,19,20]. With the principle of superposition, the CM has been developed to measure the biaxial stress distribution on a cross-section plane by integrating other stress measurement methods, such as the slitting method [21] and the XRD method [22,23]. Furthermore, the CM can be also used to measure the multiple residual stress components by multiple cuts [24].

In the present study, the contour method with multiple cuts was adopted to measure the residual stresses in a thick butt-welded joint with a partial repair weld, the residual stress maps on three cut planes were obtained and the internal stress in repair region were compared with that within the initial weld. The XRD method was also used to measure the surface stress distribution.

## 2. Welding Experiment

**Figure 1:**
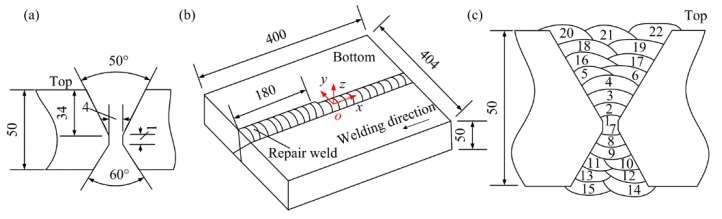
Schematic diagram of the specimen. (**a**) Groove configuration; (**b**) dimensions; (**c**) welding sequence.

Two plates with the dimensions of 200 × 200 × 50 mm were butt-welded together with the gas metal arc welding (GMAW). The groove configuration and the final dimensions of the specimen are shown in Figure 1a,b. The 1.2 mm-diameter flux-cored welding wire (CHT711HR) was used and the CO_2_ shielding gas was employed during welding. The base metal is Q345D steel with a yield strength of 392 MPa, and the yield strength of the weld metal is 543 MPa. The chemical compositions of the base metal and the weld metal are shown in Table 1.

**Figure 2:**
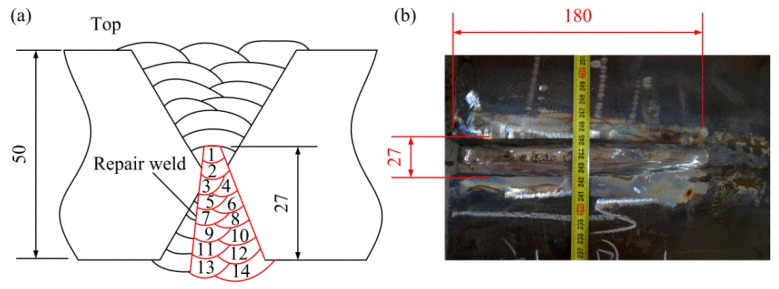
Welding sequence and dimensions of the repair weld. (**a**) Welding sequence; (**b**) dimensions.

There are 22 passes used to finish the initial weld. After finishing pass 6, the root pass (pass 1) was removed by the carbon arc gouging, and then the subsequent passes were filled. The welding sequence is shown in Figure 1c. The preheating temperature and the interpass temperature were 60 °C. After welding, part of the weld was removed from the bottom surface by carbon arc gouging to create a localized excavation and the surfaces of the excavation were ground, then the excavation was re-welded (the repair weld was introduced). The location of the repair region is illustrated in Figure 1b. The maximum depth of the excavation is approximate 27 mm, the maximum width is about 27 mm, and the length of the repair weld is 180 mm. Total 14 passes were adopted to finish the repair weld. The welding sequence and the dimensions of the repair weld are shown in Figure 2. The welding parameters employed for the original weld and subsequent repair are shown in Table 2.

## 3. Stress Measurement

### 3.1. Three-Cut Contour Method

While theoretical details for the CM can be found in the reference [10], a summary of implementation of the method is provided here: (1) cutting of the part along the plane-of-interest; (2) measuring the cut surface contours (out-of-plane displacement caused by stress relief); (3) smooth fitting the measured contours to eliminate artefacts and noise introduced by the cutting process; and (4) computing original residual stress using a fully elastic finite element (FE) analysis.

**Figure 3:**
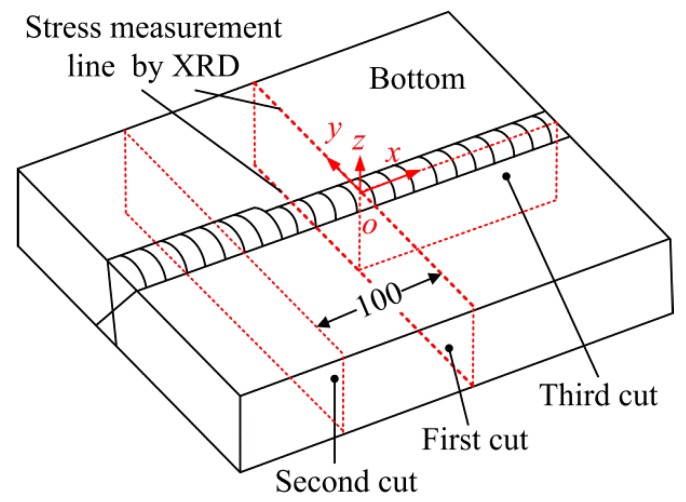
Schematic diagram of the cut planes.

The specimen demonstrated in Figure 1 contains the original weld and a partial repair weld with a length of 180 mm and a maximum depth of 27 mm. The difference between the stress in the initial weld (as-welded stress) and that in the repair region would be distinguished. In the present study, three cuts were performed sequentially on the specimen and the deformations of the cutting planes were measured, then the stress on the planes can be obtained with the contour method and the superposition-based theory. The schematic diagram of the three cut planes on the specimen are shown in Figure 3. The plane of the first cut locates at the original weld and the second cut plane locates at the middle length of the repair weld, the distance between the first cut plane and the second cut plane is 100 mm. The location of the third cut plane is the weld centerline. The stress normal to the cut plane can be obtained according to the principle of the CM [10], the longitudinal stresses on the planes of the first cut and the second cut, as well as the transverse stress on the plane of the third cut, can be obtained by the CM and the superposition theory, which is explained as follows.

**Figure 4:**
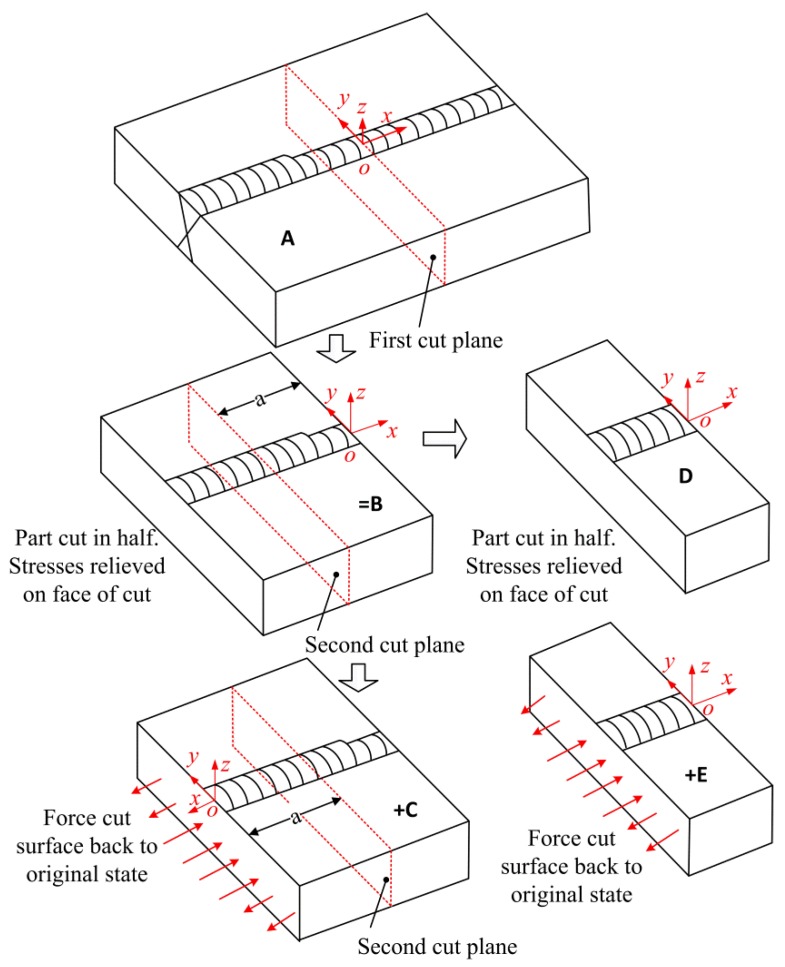
Superposition principle to reconstruct the original stresses on the plane of the second cut.

According to Pagliaro et al. [24], the theory for the superposition of stress released by the first cut and that deduced by the second cut to reconstructing the original longitudinal stress on the plane of the second cut is illustrated in Figure 4.

In Figure 4, **A** is the intact specimen containing the residual stresses that are to be measured. **B** is the half of the specimen with partially-relaxed stresses. **C** is the analytical step that starts with a stress-free half part of the specimen and then the surface created by the cut is forced back to its original flat shape. Because the stresses on the cut plane in **B** are fully released, the stresses deduced by **C** along the plane of the first cut are the original stresses on the first cut plane. Since the measured contour of the cut plane just provides the displacements in the normal direction, only the original longitudinal stress along the first cut plane can be obtained. After the second cut, **D** is the quarter-part of the specimen with the residual stress relaxation. **E** is the analytical step in which the surface created by the second cut is forced back to its original flat shape, i.e., the stresses in **E** are the remaining stresses after the first cut. The stress in **B** is given by the superposition of the stress in **D** and that in **E**. The stresses on the cut face in **D** are fully released, and the released stressed along the second cut plane caused by the first cut can be deduced by C. Therefore, the sum of **C** and **E** will give the original stresses along the plane of the second cut. As mentioned before, the normal stress component along the second cut plane (part of the original longitudinal stress) can be experimentally determined.

According to the analysis above, the original longitudinal stresses along the first cut plane and the second cut plane can be expressed as the equations

(1) $$\begin{array}{l} \sigma_{x}^{\left(A\right)}(0,y,z)=\sigma_{x}^{\left(C\right)}(0,y,z)\\ \sigma_{x}^{\left(A\right)}(-a,y,z)=\sigma_{x}^{\left(C\right)}(-a,y,z)+\sigma_{x}^{\left(E\right)}(-a,y,z) \end{array}$$

**Figure 5:**
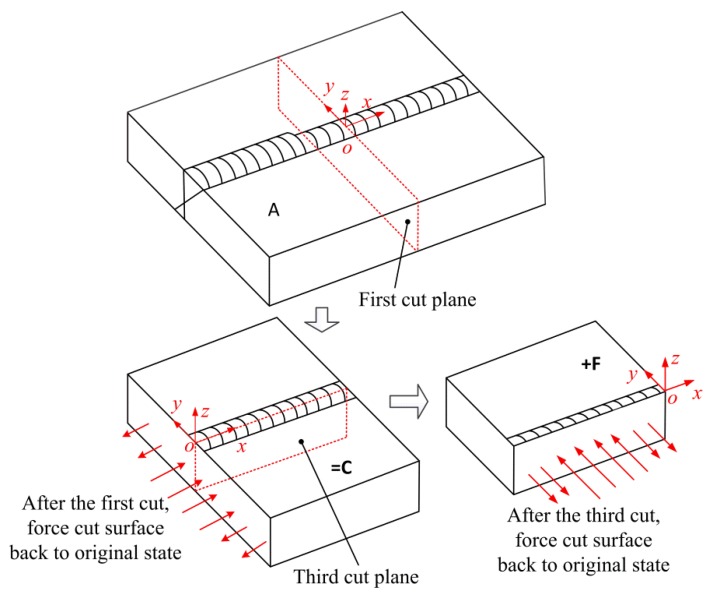
Superposition principle to get the original transverse stress along the plane of the third cut plane.

In the same way, the original stresses along the third cut plane can be obtained by superposing the stresses along the third plane in **C** and those on the third cut plane caused by forcing the cut surface back to its original state after the third cut (**F**), as shown in Figure 5. The deformation in the direction normal to the plane of the third cut can be experimentally measured. Therefore, the normal stress component along the third cut plane—i.e., the transverse stress—can be determined. The original transverse stress on the plane of the third cut can be expressed as the equation

(2) $$\sigma_{y}^{\left(A\right)}(x,0,z)=\sigma_{y}^{\left(C\right)}(x,0,z)+\sigma_{y}^{\left(F\right)}(x,0,z)$$

In the present study, each cut was performed on the Sodick ALN400Qs wire electric discharge machine (EDM, Sodick Amoy Co. Ltd., Xiameng, China) with a 250 μm diameter brass wire at a nominal cutting speed of 0.12 mm/s. The specimen was securely clamped onto the worktable of the EDM during cutting with a custom fixture as used in [11]. After cutting, the contours of the cut faces were measured using a Hexagon Global Performance coordinate measuring machine (CMM, Hexagon Metrology (Qingdao) Co. Ltd., Qingdao, China) by measuring the surface height at a grid of in-plane locations. For the contour measurement of the cut planes after the first cut, a region consisting of about 80 mm to either side of the weld center was measured with a point spacing of 1 mm to better capture the surface profile in the area of expected stress gradients, and other regions were measured with a point spacing of 2 mm. The point spacing for measuring the surfaces of the second cut was also 1 mm. The surface contour data from the two opposite surfaces were carefully aligned and averaged for each cut. Then, the averaged contour was fit to a smooth surface using bivariate splines. The residual stress on the contour plane was found with a linear elastic finite element analysis that applies the smoothed surface profile as a displacement boundary condition on the cut plane. The ANSYS commercial finite element software (Version 16.0, ANSYS Inc., Canonsburg, PA, USA) was used for the modelling and linear elastic static analysis. A geometric model was first built with the dimensions of the after-cut part, and then the eight-node brick elements (Solid185) were used to mesh the model. For the first cut and second cut, node spacing of 1 mm on the cut face was used and that in the direction normal to the cut face was 2 mm. For the third cut, the node spacing of 2 × 1 mm was employed on the cut face and that was 2 mm in the direction normal to the cut face. To avoid rigid body motion, the model was constrained at two corners of the model with three additional displacement constraints leading to no reaction forces as used in the reference [10]. The model used an elastic modulus of 200 GPa and a Poisson’s ratio of 0.30.

### 3.2. XRD Method

Before the cut of the contour method, the stress distributions on the top and bottom surfaces were measured by the XRD method to verify the measured results by the contour method. An automated portable diffractiometer (Proto iXRD, Proto Manufacturing Ltd., Oldcastle, Canada) with the sin2ψ method employed. The X-ray was generated from a chromium tube (20 kV, 10 mA) with the beam diameter of 1 mm. The diffraction plane was the {211} crystallographic plane. The measuring locations of the surface stress are shown in Figure 3.

## 4. Results and Discussions

### 4.1. Measured Results by the Contour Method

**Figure 6:**
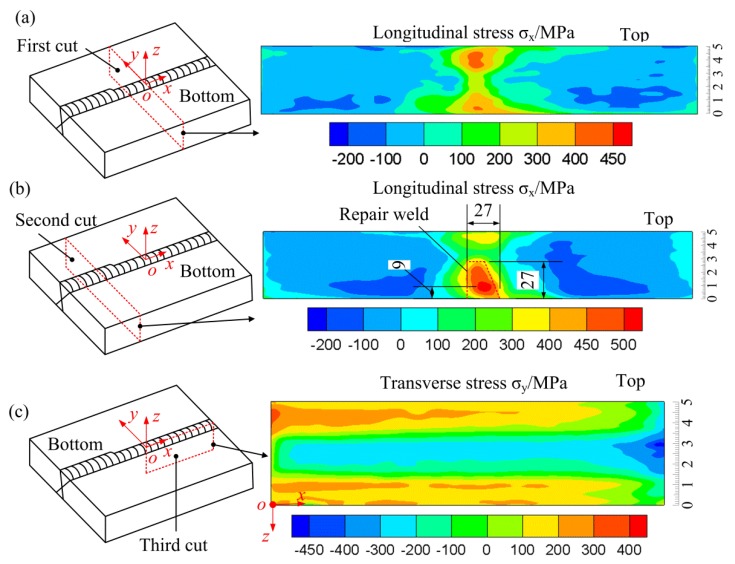
Stress distributions along the three cut planes. (**a**) Longitudinal stress along the plane of the first cut; (**b**) longitudinal stress along the plane of the second cut; (**c**) transverse stress along the plane of the third cut plane.

Based on the single-cut contour method and the superposition principle of the multi-cut contour method, the residual stress maps at different locations can be obtained, as shown in Figure 6.

According to the investigation on the repair weld in the girth weld carried out by George and Smith [7], at a location 25 mm beyond the end of the repair weld (in the original weld), the hoop residual stress is found to be essentially the same as the as-welded stress as if no repair had been introduced. In the present study, the distance between the end of the repair weld and the first cut plane (within the original weld) is about 20 mm. In addition, the end of the repair weld is very shallow, therefore the longitudinal stress on the first cut plane measured by the CM should not be affected by the repair weld and it should be the as-welded longitudinal stress. Accordingly, the longitudinal stress on the first cut plane (as-welded stress) can be compared with that on the second cut plane (stress after repair) and the difference between them can be investigated.

Figure 6a shows the longitudinal stress on the plane of the first cut (as-welded stress within the original weld). As shown in Figure 6a, tensile longitudinal stress is present within the weld zone with small compressive longitudinal stress occurring in the region outside the weld zone to balance the tensile stress in the weld zone (the value of compressive stress ranges from 0 to −200 MPa). The longitudinal stress near the top and bottom surfaces is larger than that in the interior. The peak value of the tensile longitudinal stress reaches about 440 MPa appearing at the location about 8 mm depth beneath the top surface. Near the bottom surface, a maximum longitudinal stress of about 410 MPa is present at the location of 5 mm depth. Figure 6b illustrates the longitudinal stress in the partial repair weld along the plane of the second cut. It shows the similar distribution as that in the initial weld, large tensile longitudinal stress occurs in the weld zone and small compressive stress appears outside. While in the repair weld (the bottom part of the weld zone), the peak longitudinal stress is about 530 MPa occurring at the location of about 9 mm beneath the bottom surface of the repair weld, which is approximately the yield strength of weld metal at room temperature and it is also larger than that in the original weld at the same location (on the plane of the first cut). Since the repair beads were deposited under strong restraints (the two plates were already welded together), the longitudinal stress increased in the repair region. Compared with Figure 6a, the longitudinal stress within the repair weld as a whole in Figure 6b is relatively larger than that in the original weld, while the stress in the region above the repair weld is smaller than that in the same region on the plane of the first cut. This is most probably due to the heat treatment effect introduced by the repair weld, which resulted in a stress decrease in the region. From Figure 6b, it can be concluded that the introduction of the partial repair weld with a limited depth raises the tensile longitudinal stress in the repair region to a value close to the yield strength and causes a decrease in the longitudinal stress in the region above the repair weld.

Figure 6c demonstrates the transverse stress distribution at the weld centerline along the plane of the third cut. A distinct self-equilibrated transverse stress distribution is present in the figure with tensile stress occurring towards the top and bottom surfaces and compressive stress presenting in the interior. This kind of stress distribution is the common type of the as-welded transverse at the weld centerline for butt-welded joints by multipass arc welding with the single V groove or double V-type groove as demonstrated by Kartal et al. [25], Liu et al. [11,26], and Smith et al. [27].

### 4.2. Comparison of the Results by the Contour Method and XRD Method

**Figure 7:**
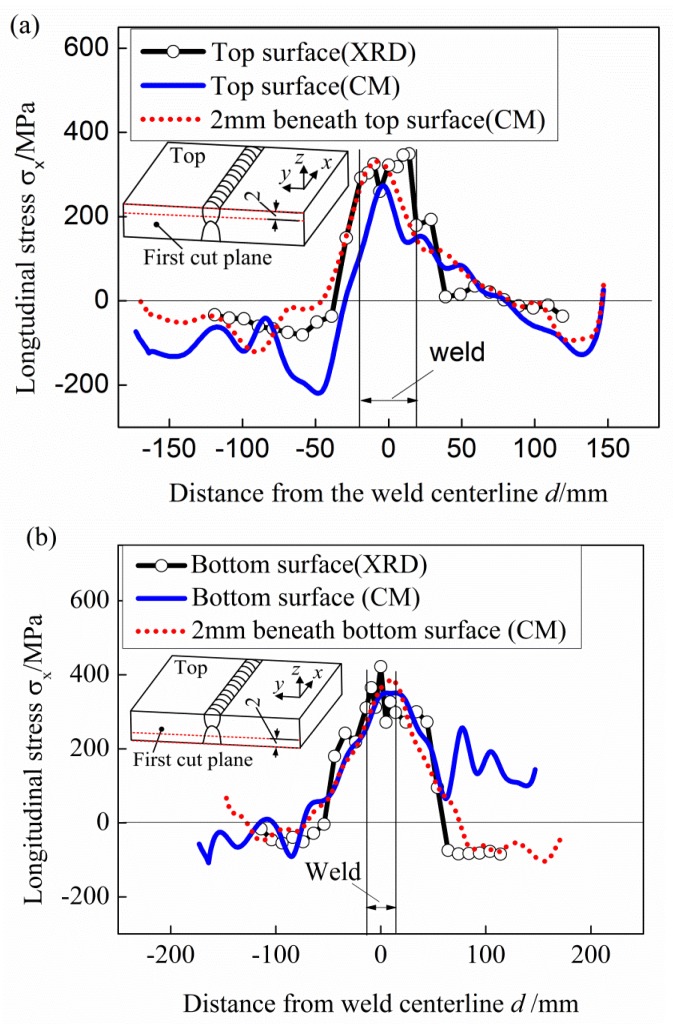
Comparison between the results measured by XRD and CM. (**a**) Top surface and 2 mm beneath the top; (**b**) bottom surface and 2 mm beneath the bottom surface.

The measured surface stresses by the XRD method are compared with those measured by the CM along the lines on the surfaces and lines with a distance of 2 mm beneath the surfaces, as shown in Figure 7.

There are additional errors in the displacement measurement near the edges of the cut plane [10,14,28], caused by machining irregularities on the edge and the spherical tip of the CMM (2 mm in diameter in the present study) going slightly past the actual edge of the part. Therefore, the surface stresses obtained through the CM method are generally less accurate when compared to the XRD. As shown in Figure 7, the discrepancies between the longitudinal stress distribution on the top and bottom surfaces obtained by the CM and the XRD are large, especially on the top surface. However, the stress magnitudes and distributions along the lines with 2 mm distance beneath the surfaces match quite well with the surface stresses measured by the XRD. Since the change of longitudinal stress within 2 mm-depth surface layer is not significant, it is believed that the measured results obtained by the CM is reliable and verified by the XRD.

### 4.3. Stress Distribution at Different Locations

**Figure 8:**
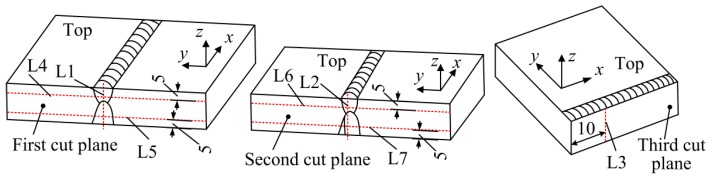
Schematic diagram of the locations of the stress evaluation lines.

To clear analyze the stress distribution on each cut plane, seven lines are selected from the cut planes and the stresses along these lines are compared. The locations of these seven lines are shown in Figure 8. Line L1, line L2, and line L3 are used to evaluate the through-thickness stresses at the weld centerline on the cut planes. Line L4 and line L6 are the lines with a distance of 5 mm from the top surface of the cut planes, and line L5 and line L7 are those with a distance of 5 mm from the bottom surface of the cut planes. Uncertainty of CM tends to be larger at the boundaries of the cut planes due to limits on EDM cutting and contour measurement as mentioned before, the line L3 is selected on the plane of the third cut with a distance of 10 mm from the cut edge.

**Figure 9:**
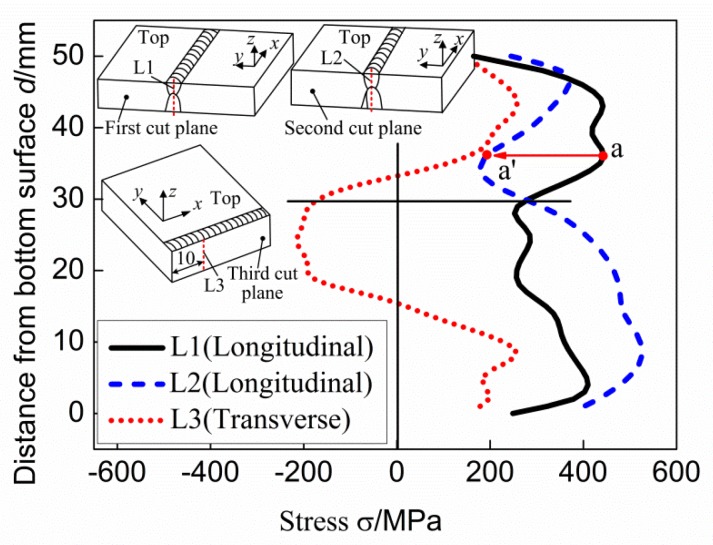
Stress variations along the thickness.

Figure 9 shows the stress variations along line L1, line L2, and line L3. The stress along the line L1 is the through-thickness as-welded longitudinal stress at the weld centerline, and that along the line L2 is the through-thickness after-repair longitudinal stress at the weld centerline. From Figure 9, it can be seen that the after-repair longitudinal stress along the thickness at the weld centerline (longitudinal stress along the line L2) decreases within the top region of the weld (0–20 mm distance from the top surface) and increases within the repair region (0–30 mm distance from the bottom surface) as compared with the as-welded longitudinal stress (stress along the line L1). The introduction of the repair has increased the peak tensile longitudinal stress in the repair region by 115 MPa. For example, the peak tensile stress is about 410 MPa for the original weld near to the bottom surface, and in the repair weld the peak tensile longitudinal stress is raised to about 525 MPa. In addition, the peak tensile longitudinal stress in the top region of the original weld beyond the repair is 440 MPa, occurring at the location of 14 mm distance from the top surface (point a in Figure 9), and stress becomes 180 MPa at the same location in the region above the repair (point a’ in Figure 9). It means that the maximum longitudinal stress reduction induced by the partial repair in the region above the repair weld can reach 260 MPa.

The transverse stress along the thickness in Figure 9 (along Line L3) demonstrates a self-equilibrated distribution, which is tensile towards the top surface, dropping into compression in the central region and returning to tension towards the bottom surface. 

The residual stresses are those stresses which are retained within a body when no external forces are acting and therefore they are self-equilibrating inside the free body [1]. The welding residual stress distributions in different directions are completely different as shown in Figure 9. This is caused by the different restraints acting in the directions. Welding residual stresses are mainly generated due to the thermal contraction of the weld metal and the adjacent heated parent during cooling. Longitudinal shrinkage of the weld is strongly resisted by the parts being joined, so high tensile residual stresses are generated throughout the thickness of the weld. The longitudinal stress is self-equilibrating along the transverse direction as shown in Figure 6a,b. The transverse shrinkage of the final weld passes on the upper and lower surfaces of the plate is resisted by the passes deposited previously, so tensile residual stresses are generated near the upper and lower faces, balanced by compression at mid-thickness, as shown in Figure 6c and Figure 9.

**Figure 10:**
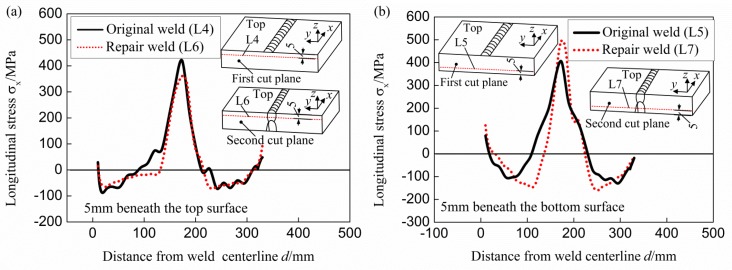
Stress variation along lines with 5 mm distance from the top and bottom surfaces. (**a**) Near the top surface; (**b**) near the bottom surface.

Figure 10 shows the stress variation along lines with 5 mm distance from the top and bottom surface on different cut planes (in the original weld and the repair weld). As demonstrated in Figure 10a, the longitudinal stress distribution along line L4 (original weld beyond the repair weld near to the top surface) is almost the same as that along line L6 (original weld above the repair weld), except the smaller peak tensile stress along line L6 induced by the repair weld. While the stress distribution along L5 (original weld beyond the repair weld, near to the bottom surface) is a little different from that along line L7 (repair weld near the bottom surface) as demonstrated in Figure 10b, the tensile stress width along line L5 (original weld) is larger than that along line L7 (across repair weld) and the peak tensile longitudinal stress along line L5 is smaller than that along line L7. It can be concluded that the introduction of partial repair weld does not affect the stress distribution trend in the original weld (no matter beyond or above the repair weld), and has slight effect on the tensile stress distribution width in the repair region with significant effect on the peak value of the longitudinal stress in the original weld above the repair weld.

## 5. Conclusions

The stress distributions in a thick welded specimen with a partial repair weld were measured with the three-cut contour method. The longitudinal stress maps in the original weld and the repair weld were obtained and the transverse stress map at the weld centerline in the original weld was also acquired. The variation of the longitudinal stress before and after the repair was investigated. The main findings can be concluded as follows:

(1) The contour method with multiple cuts based on the superposition principle can be used to get stress distribution maps at different cut locations. The measured longitudinal stress through the CM along the lines with 2 mm distance beneath the top and bottom surfaces can be verified by the surface stress measured by XRD.
(2) The partial repair weld has a great effect on the magnitude of as-welded longitudinal stress in the repair region and the region above the repair. Comparing with the stress in the original weld, the longitudinal stress increases significantly throughout the entire repair region and that decreases distinctly in the region above the repair weld due to the heat treatment effect.
(3) The introduction of the repair weld raises the peak through-thickness longitudinal stress by about 115 MPa at the weld centerline in the present study, resulting in a peak tensile longitudinal stress close to the yield strength of weld material at room temperature. A maximum longitudinal stress reduction of about 260 MPa is induced by the repair weld in the region above the repair weld in the present study.
(4) The introduction of partial repair weld does not affect the stress distribution trend in the original weld (whether it is beyond or above the repair weld), and it has slight effect on the tensile stress distribution width in the repair region.

## Figures and Tables

**Table 1:** Chemical compositions of the base metal and weld metal (wt %).

Base/Weld Metal	Chemical Composition						
	C	Mn	Si	S	P	Cr	Fe
Q345D (base metal)	0.17	1.52	0.22	0.009	0.023	-	Balance
Weld metal	0.069	1.30	0.36	0.006	0.019	0.037	Balance

**Table 2:** Welding parameters.

Weld	Pass Number	Voltage/V	Current/A	Welding Speed/mm·min^−1^
Initial weld	1–3	30–32	180–220	188–220
	4–13	30–32	230–270	200–300
	14–15, 20–22	29–30	180–220	180–200
	16–19	31–33	240–260	230–300
Repair weld	1–3	30–31	210–250	520–580
	4–12	30–31	220–250	250–400
	13–14	30–31	220–260	300–330
